# Parents’ Knowledge and Attitudes Toward Pediatric Ophthalmic Disorders in Saudi Arabia: A Cross-Sectional Study

**DOI:** 10.3390/pediatric16040077

**Published:** 2024-10-17

**Authors:** Saja Radhi G. Alanazi, Haneen Wadi H. Alanazi, Wasan Ghathwan Alanazi, Nawal Surhuj Q. Alanazi, Dareen Owaid B. Alenezi, Maisa Al-Sweilem, Maram Hassan Alqattan, Iftikhar Lafi N. Alanazi, Jumanah Mohammed Tirksstani, Reem Saeed AlSarhan, Saleh Ghulaysi, Hanan A. Elgendy, Manal S. Fawzy

**Affiliations:** 1 Faculty of Medicine, Northern Border University, Arar 91431, Saudi Arabia; sajaradhi1@gmail.com (S.R.G.A.); haaa.wa1009@gmail.com (H.W.H.A.); wasannnn123459@gmail.com (W.G.A.); dr.nawal.alsultani24@gmail.com (N.S.Q.A.); darinalanzi999@gmail.com (D.O.B.A.); eftekhar.1420@hotmail.com (I.L.N.A.); 2 Ophthalmology, General Directorate for Health Investment, Ministry of Health Branch North Zone, Arar 73311, Saudi Arabia; dr.maisams@gmail.com; 3 College of Medicine, King Faisal University, Al-Ahsa 31982, Saudi Arabia; mramalqtan@gmail.com; 4 College of Medicine, King Saud bin Abdulaziz University for Health Sciences, Jeddah 11481, Saudi Arabia; tirksstani043@ksau-hs.edu.sa; 5 College of Medicine, Princess Nourah Bint Abdulrahman University, Riyadh 11671, Saudi Arabia; reem.saeed29@gmail.com; 6 Faculty of Medicine, Jazan University, Jazan 45142, Saudi Arabia; salehghulaisy@gmail.com; 7 Department of Anatomy, Faculty of Medicine, Northern Border University, Arar 91431, Saudi Arabia; hanan.eljendy@nbu.edu.sa; 8 Unit of Medical Research and Postgraduate Studies, Faculty of Medicine, Northern Border University, Arar 91431, Saudi Arabia; 9 Department of Biochemistry, Faculty of Medicine, Northern Border University, Arar 91431, Saudi Arabia

**Keywords:** Saudi Arabia, parents, eye disease, children, awareness, visual health education

## Abstract

Recognizing pediatric eye health issues at an early stage, along with ensuring that parents are well informed, is crucial. This study measures parents’ knowledge and perspectives on pediatric ophthalmic disease. The study utilized a cross-sectional design, and participants included Saudi parents of children residing in Saudi Arabia. Data were collected using a pre-validated self-administered questionnaire with a scoring system. Sociodemographic characteristics and factors associated with knowledge/attitude were collected and analyzed. Initially, 425 respondents participated in this study. Excluding the participants whose work was related to healthcare and those with incomplete data yielded 370 responses on which the subsequent analyses were performed. The analysis revealed that only half of the parents recognized the need for annual eye examinations for children. Most of them showed gaps in knowledge regarding the frequency of routine eye exams and indicators of visual problems. Notably, parents with good/excellent knowledge were more proactive in their eye care practices, such as adherence to recommended eye examination schedules and accepting corrective measures like glasses for common conditions such as refractive errors and amblyopia. However, parental willingness to permit surgical interventions did not correlate significantly with their level of knowledge, signaling the influence of other factors. In conclusion, this study underscores the need for enhanced public health education to improve parental awareness of pediatric eye diseases in Saudi Arabia. Given the link between knowledge and proactive eye health practices, targeted interventions should distribute comprehensive, culturally sensitive information accessible to all demographics.

## 1. Introduction

Pediatric eye conditions represent a significant public health concern, with profound implications for the developmental potential and quality of life of affected children [1,2]. Within the unique cultural and demographic landscape of Saudi Arabia, characterized by a predominantly young population, diverse customs, and specific health beliefs, examining parental awareness and attitudes toward these diseases is particularly compelling [3,4]. The healthcare system in Saudi Arabia is structured around a model that emphasizes both public and private services, featuring a network of primary healthcare centers and specialized hospitals [5]. However, disparities in access to high-quality medical resources exist between urban and rural areas, which can affect the availability of pediatric eye care services, further complicating the management of these conditions [6].

The cultural norms in Saudi Arabia may influence perceptions of health and healthcare practices, including the stigma surrounding eye conditions and the use of corrective eyewear [7]. Furthermore, the rapid urbanization and socioeconomic changes in the region contribute to varying levels of access to healthcare services and information, directly impacting how pediatric eye health is prioritized [8]. The strong family ties and community networks in Saudi society can act as facilitators or barriers in disseminating health information, thereby affecting parents’ attitudes toward seeking timely care for their children’s eye health [9].

In terms of specific eye diseases, local epidemiological studies have highlighted that the most common pediatric eye conditions in Saudi Arabia include refractive errors (such as myopia and hyperopia), strabismus (crossed eyes), and infections of the conjunctiva and cornea [10,11,12,13]. Research indicates that refractive errors are particularly prevalent among school-aged children, often exacerbated by factors such as screen time and lack of outdoor activities [14]. These conditions may exhibit higher prevalence rates compared to some other countries, owing to lifestyle and environmental factors [14,15].

Currently, there is no standardized age-related screening program for pediatric eye conditions across Saudi Arabia, although some initiatives exist in certain regions, particularly within school health programs [16]. Screening generally occurs through community outreach, health fairs, and school-based assessments. However, there are significant barriers that hamper regular routine screenings, including variability in healthcare access, cultural beliefs affecting health-seeking behavior, and a lack of awareness about the importance of early detection among parents and caregivers [17]. These factors can result in missed opportunities for timely intervention.

It is crucial to understand the global burden of pediatric eye conditions and the specific local variations to devise interventions appropriately tailored to the Saudi Arabian population [1]. Globally, an estimated 19 million children are affected by visual impairments, many of which are preventable or treatable, according to the World Health Organization [18]. Such impairments go beyond affecting immediate health, potentially hindering educational success and causing long-term challenges in social and economic independence. The emphasis, therefore, on early detection and prompt management cannot be understated [2,3,5]. However, routine screening practices can have their pitfalls, such as false positives, unnecessary anxiety, and overutilization of resources, which may undermine overall efforts to improve pediatric eye health. Understanding these nuances is essential for creating effective screening programs and ensuring they align with the needs of the population [19].

In Saudi Arabia, the dynamics of pediatric eye disease prevalence are shaped by social beliefs, healthcare infrastructure quality, and the disparities between regions; it is within these parameters that parental awareness becomes crucial for the timely and effective management of these conditions [20,21,22,23]. Without appropriate attention, there is a risk that these diseases could progress to permanent vision loss [24,25].

The broader societal impact of neglected pediatric eye conditions is substantial; it encompasses not only the individuals directly affected but also the national economy. A population with impaired vision faces challenges in labor productivity, potentially leading to increased dependence and diminished quality of life [26]. Tackling this issue is thus an essential element of national strategy, aligning closely with the goals of Saudi Arabia’s Vision 2030 by fostering social and economic progress [11].

Cultural norms and health beliefs shape parental attitudes and choices regarding their children’s eye health [27,28]. Addressing and dispelling the stigma around eye conditions and their treatments, such as using corrective eyewear, is essential to encourage proactive eye care practices [5,7].

Local epidemiological studies have highlighted a range of prevalent eye conditions, presenting distinct patterns across different Saudi regions [29,30]. These insights point to the necessity of tailored awareness programs and intervention strategies. Despite the potential to mitigate visual loss through informed and early interventions, research indicates that parental awareness in key Saudi areas is varied and inconsistent [29,30,31].

This study aims to bridge the crucial gap in understanding by assessing the level of knowledge and attitudes of parents toward pediatric eye diseases across various Saudi regions. The objective is to pinpoint critical areas needing educational and healthcare initiatives and to guide policymakers and health professionals toward optimal strategies to enhance eye health awareness among parents. Our ultimate goal is to initiate a positive change that guarantees timely interventions and decreases the incidence of preventable childhood blindness.

## 2. Materials and Methods

### 2.1. Study Design

This cross-sectional and observational study spanned various regions within Saudi Arabia from 20 February to 31 March 2024. Data were collected using a questionnaire that participants completed on their own. The Northern Border University’s Local Bioethics Committee in Arar, Saudi Arabia, granted ethical approval for this investigation (approval ref: 17/24/H, issued on 19 February 2024).

### 2.2. The Target Study Participants

To determine the necessary number of participants for robust study results, we used an online sample size calculator provided by Raosoft (Raosoft Inc., Seattle, WA, USA). The calculation indicated a requirement for 377 individuals to achieve a 95% confidence level with a margin of error set at 5.05%. Once we set our participant criteria to include only Saudi parents of children under 18 years residing in Saudi Arabia who consented to join the study, we identified 370 participants with complete data who met these conditions.

### 2.3. Study Sampling and Data Collection

A convenience sampling method was applied for participant recruitment. The study was advertised through different social media platforms, such as WhatsApp, Facebook, and X, by sending the link to the prepared Google form with an introductory section explaining the aim of the study and confirming data confidentiality and anonymity. The respondent could only fill in the form after giving his/her consent to participate in the study. For this research, we utilized an Arabic questionnaire completed by the participants themselves, which was created concerning Almogbel et al.′s survey [6] and was adopted in our study after obtaining the original authors’ approval. The English version of the questionnaire underwent a translation process to Arabic by two translators proficient in both languages. Following this initial translation, a panel of language experts and translators reviewed the Arabic version to rectify discrepancies. A subsequent translation back to English (reverse translation) was executed to verify the accuracy of the questionnaire, and the integrity of the research instrument remained intact. To further refine the tool, prior to the commencement of the official data gathering, a pilot group of 10 individuals received the questionnaire. Their insights led to a simplification of the medical jargon and adjustments in the structure of specific questions within the survey. The survey comprised four sections with a total of 32 structured questions. The initial section focused on demographic information, gathering ten inquiries about sex, age, education level, job status, income ranges, and residence area. The subsequent portion featured five items to gauge parents’ knowledge of eye care essentials. This was followed by the third division, with 11 questions dedicated to understanding parental awareness of various pediatric eye conditions. The concluding quarter of the questionnaire presented six items concerning practices related to eye care maintenance.

### 2.4. Consideration of Societal Structure

In designing the study, we specifically acknowledged the socioeconomic structure of Saudi society, which features significant unemployment rates (nearly 40% of citizens) and a prevalence of low-income households, alongside a high level of educational attainment (over 70% with university degrees) [32]. We aimed to recruit a diverse sample that reflects these community dynamics to ensure that our findings would be representative of different socioeconomic backgrounds. Participants were encouraged to share their job status and income levels in the demographic section, allowing us to analyze how these factors may influence awareness and attitudes toward pediatric eye health.

### 2.5. Statistical Analysis and Coring System

Data were analyzed using SPSS (IBM Corp., Armonk, NY, USA), version 22. Descriptive statistics were employed to summarize the demographic characteristics of the study population. Categorical data were presented in frequencies and percentages. In assessing knowledge, a scoring system was used where each correct response earned one point. Separate points for accurate knowledge responses were collected to gauge the overall understanding of pediatric eye diseases. A score of 75% or higher of the maximum attainable points was categorized as excellent knowledge. Those with scores from 50% up to 74% fell into the good knowledge range, whereas scores below 50% indicated inadequate knowledge [20]. For simplification, the good and excellent knowledge classifications were merged into one knowledge category to facilitate running the statistical analysis. The chi-square test was utilized for categorical variables to compare the awareness levels across different demographic groups, and the exact probability test was applied if there were small frequency distributions. The logistic regression analysis was applied to determine the predictor most associated with parents’ awareness regarding pediatric eye diseases, controlling for various demographic factors. The level of statistical significance was set at a *p*-value < 0.05. All reported *p*-values were two-tailed.

## 3. Results

### 3.1. Demographic Characteristics of the Study Participants

Table 1 illustrates the sociodemographic characteristics of the initial 425 respondents who participated in the study. The sample was predominantly female (85.6%) and young adults, with 290 (68.6%) falling within the 18–40-year-old range. The participants were distributed across different regions of Saudi Arabia, with the highest representation from the Northern area (57.4%) and the lowest from the Eastern region (6.1%). The educational level among the study group varied, indicating generally high educational attainment; many respondents were university graduates or held higher credentials (Master’s or Doctorate), making up 73.9%. About 12.9% of the initial study participants had occupations related to eye health. Hence, they were excluded from the subsequent analyses to eliminate potential bias and ensure the applied assessment of parental knowledge regarding pediatric eye diseases was based on layperson understanding. This measure left us with a total of 370 responses that were included in the further assessment.

### 3.2. Parental Knowledge Regarding Pediatric Eye Care

Table 2 represents a comprehensive overview of parental awareness regarding pediatric eye care among 370 respondents. Most parents (65.4%) reported that their children did not suffer from any eye diseases. Nevertheless, a notable proportion of children reported having common eye conditions. Refractive errors, such as nearsightedness or farsightedness, were the most prevalent, affecting 16.8% of respondents’ children, followed by lazy eye (amblyopia) at 7.6%. Other less common conditions included strabismus at 1.9%, congenital cataracts at 0.8%, and eye allergies reported by only 0.5%. 

Concerning the impact of vision problems on education, the vast majority of parents who acknowledged eye conditions in their children (33%) indicated that their children with vision problems continued to attend school. However, a small fraction (1.6%) had children who did not attend school due to vision problems. The reasons for school non-attendance included concerns about inadequate learning due to poor vision (10%), fear of bullying by peers (5.1%), fear of a lack of adaptation (4.3%), wearing glasses (0.8%), a singular report of eye strain (0.3%), and 14.1% did not know the cause.

When surveyed about their sources of information on eye diseases, many parents reported using a combination of mentioned media tools (43%), suggesting a diverse approach to seeking health information. The internet stood out as a source of 24.9%, underscoring its importance as a critical avenue for information dissemination. A noticeable reliance was also placed on social media (17.5%) and relatives and friends (12.2%). However, traditional media such as television seemed to be a less common information source, mentioned by only 2.4%.

Regarding the preferred methods for receiving information about children’s eye health, about 55% favored receiving information through a combination of all suggested means. Direct communication from doctors was preferred by 24%. Meanwhile, other methods, such as awareness campaigns (5.4%), social media (10.8%), and the internet (3.8%), were less favored. However, traditional broadcasting means like broadcasting boards (0.5%) and television (0.3%) were the least preferred, confirming the trend towards digital media for health information dissemination.

### 3.3. Parental Knowledge of Pediatric Eye Conditions

Table 3 displays the results concerning parental knowledge about various pediatric eye conditions within the surveyed group (*n* = 370). About half of the participants (52.7%) correctly defined amblyopia by its clinical definition, yet nearly one-third were unfamiliar with it. Misconceptions were notable, with some incorrectly attributing amblyopia to screen time. Only a minority of parents could identify standard causes like refractive errors (14%) and strabismus (13%), and treatment awareness was equally diverse, with patching being the most acknowledged (18%), but over a third unaware of treatment options at all. However, 23% of parents did not realize the benefit of early treatment intervention.

Knowledge of cataracts and glaucoma was less prevalent, with less than a quarter accurately defining each, and many were unaware entirely. While about two-thirds of participants were aware that children could develop cataracts and the associated risk of blindness, over half (56.8%) were uncertain about the impact of glaucoma on children.

In preventive care, half of the surveyed parents recommended annual eye exams for children, yet there is evidence of a reactive approach to eye health, with 13.5% waiting for visual issues to manifest before seeking professional advice. While some suggested eye exams at lengthier intervals, about 16.7% did not know the recommended frequency, suggesting a need for health education.

Concerning corrective measures, most families did not have children wearing glasses; yet, among those who did, correcting refractive errors was the primary reason. A small percentage did not understand the exact reason behind their children’s use of glasses, implying a need for better communication from professionals regarding eye health treatments. Interestingly, very few (16%) had considered contact lenses, possibly pointing to a preference for glasses or a lack of awareness of these lenses as another option for children.

Also, Table 3 demonstrated that the willingness to pursue surgical solutions for eye problems was high among parents (81%), with the vast majority open to the idea, though some hesitated due to concerns about risks and the cost involved. It shows a readiness to engage with more invasive treatments, although support and information might be necessary to alleviate fears and educate about potential barriers.

### 3.4. Overall Parents’ Knowledge Level of Pediatric Ophthalmic Diseases

**Figure 1:**
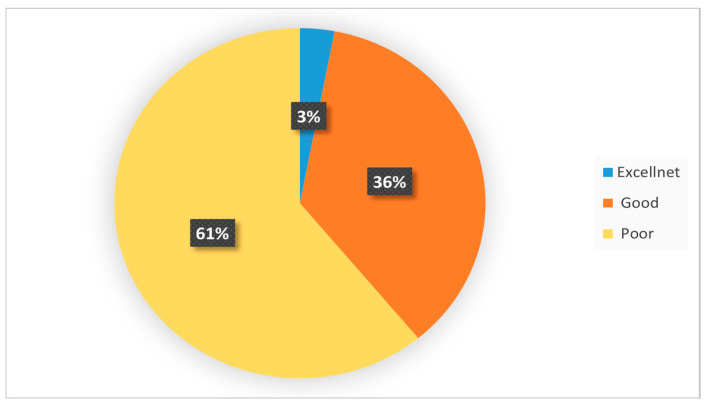
The overall parents’ knowledge level of pediatric ophthalmic diseases. Data are presented as proportions (%).

An analysis of the collected data indicates that only 3.2% of parents demonstrated an “Excellent” level of knowledge regarding pediatric eye conditions. Meanwhile, 36% of respondents displayed a “Good” level of knowledge. Parents in this category showed a reasonable understanding of pediatric eye diseases, with perhaps some gaps in knowledge or misconceptions. However, two-thirds of the participants (60.8%) fell into the “Poor” knowledge category (Figure 1).

### 3.5. Factors Associated with Parents’ Knowledge Regarding Pediatric Eye Diseases

Upon investigating the various factors that could be associated with parents’ level of knowledge regarding pediatric ophthalmic diseases, having a child with a history of eye disease showed a significant association with the parents’ knowledge level (*p* = 0.0001). Parents of children with amblyopia or refractive error demonstrated significantly higher levels of good/excellent awareness compared to those having children without ophthalmic diseases (Table 4).

Employing logistic regression analysis to control the confounders yielded the same results (Table 5). It confirmed that a child with an eye problem in the family stands out as a significant predictor for the parents having excellent or good knowledge (*p* = 0.003).

### 3.6. Relationship between Parents’ Knowledge and Their Practices Regarding Pediatric Eye Diseases

Table 6 depicts the correlation between the overall knowledge level of parents about pediatric eye diseases and their corresponding practices. A statistically significant correlation was observed between parents’ knowledge levels and the frequency of eye exams for their children. Regarding recognizing specific signs as prompts to seek professional eye care for their child, no significant difference was noted between parents with varying levels of knowledge. However, responses indicated that looking for multiple signs was quite comparable, found in 64.9% of parents with poor knowledge and 63.4% with good/excellent knowledge. The findings also indicate a significant relationship between the level of knowledge and the practice of children wearing glasses. A higher proportion of parents with good or excellent knowledge reported their children wearing glasses for refractive errors (37.2%) compared to parents with poor knowledge (20.4%). The trend was similar for lazy eye, with more knowledgeable parents reporting their children wearing glasses (14.5% vs. 7.1%).

Concerning the acceptance of children wearing contact lenses, more knowledgeable parents were likely to accept this practice (23.4%) than those with less knowledge (11.6%). However, opinions on allowing children to have necessary eye surgery did not significantly correlate with the level of knowledge.

## 4. Discussion

This cross-sectional study evaluated Saudi parents’ knowledge and attitudes toward pediatric ophthalmic diseases. The findings reveal variable knowledge and a generally proactive attitude among parents regarding pediatric eye health. However, significant knowledge gaps were identified, underscoring the need for improved public health initiatives and education to further parental understanding and engagement in this area.

Sociodemographic findings provide a comprehensive setting for understanding the context of pediatric ophthalmic disease knowledge among Saudi Arabian parents. The diverse, albeit predominantly young (18–40, 68.6%), and highly educated (74%) demographic points to a population potentially receptive to health education and intervention programs. However, the disparity in employment status and income levels could indicate varying access to healthcare resources and information, which may affect attitudes toward pediatric eye health [33]. Recognizing these variables is crucial in developing targeted public health strategies to enhance awareness and promote preventative eye care in children across the different regions of Saudi Arabia.

Our results indicate that only a small fraction of participants exhibited excellent knowledge of pediatric eye conditions, and most participants fell into the poor knowledge category. This disparity points not only to the preponderance of insufficient knowledge but also the urgency of deploying educational resources and campaigns across the community regardless of demographics. Surprisingly, factors such as parental sex, age, geographical location, educational level, and socioeconomic status did not significantly correlate with the knowledge level, suggesting that current educational strategies might not be effectively targeting or engaging these diverse parent groups. These findings are more or less similar to those of others who explored the same correlation across different regions in Saudi Arabia (Table 7) [9,20,22,23,34,35,36,37,38].

It is noteworthy that the parents who were more knowledgeable about pediatric eye diseases demonstrated good practices; they adhered to more frequent routine eye examinations and were more amenable to using corrective measures such as glasses and contact lenses. This supports the theory that education can positively influence health behaviors [39,40].

One striking result was the correlation between parental knowledge levels and their children’s use of glasses for refractive errors and amblyopia, indicating that informed parents are more proactive in seeking treatment. Notably, employing logistic regression analysis confirmed that the presence of a child with an eye problem in the family is a significant predictor of parents demonstrating excellent or good knowledge about pediatric eye diseases. This reinforces the importance of direct experiences with eye health issues in shaping parental understanding and engagement. Nonetheless, willingness to permit surgical interventions did not significantly affect knowledge levels, suggesting that other factors such as cultural beliefs, fear of outcomes, or socioeconomic conditions could be more influential in such decisions [41].

Moreover, the source of information about pediatric eye diseases did not significantly affect knowledge levels. This could indicate that the material available through these channels, which includes social media, television, and healthcare providers, is either not sufficiently comprehensive or not effectively communicated. An emphasis on the direct experience with a child’s eye condition highlighted the need for public health strategies to clarify eye conditions and their implications [42].

Collaborative efforts to improve the parental understanding of pediatric eye diseases are essential [43]. This study lays the groundwork for future interventions to empower parents to make informed decisions regarding their children’s eye health, ultimately contributing to the prevention, early detection, and effective management of pediatric ophthalmic diseases in Saudi Arabia.

## 5. Study Limitations

This study has several limitations. First, there is potential self-selection bias, as participants who are more interested in health topics may be more likely to participate, potentially skewing the sample toward a higher level of awareness. Moreover, our assessment was based on self-reported data, which may not accurately reflect actual knowledge or behaviors. Additionally, the overlap of participants from the same family in evaluating parents’ knowledge and attitudes could influence our findings. We also did not collect information regarding the occupations of family members, such as partners, who may work in the eye health field. This oversight could introduce further bias, as the knowledge and attitudes of family members in this sector might affect the participants’ responses. Furthermore, we did not gather data on the number of children or their ages for each participant. Future studies are recommended to implement stricter criteria for participant selection and to consider the occupational backgrounds of family members, as well as to collect demographic information about the participants’ children. These efforts will enhance the validity of the findings across studies.

## Figures and Tables

**Table 1:** Sociodemographic characteristics (total responses = 425).

Sociodemographic Characteristics	Category	Frequency and Proportion, *n* (%)
Sex	Male	61 (14.4%)
	Female	364 (85.6%)
Age	18–40 years	290 (68.6%)
	>40–60 years	129 (30.4%)
	60 years>	6 (1.4%)
Living area	Northern	244 (57.4%)
	Southern	41 (9.7%)
	Western	75 (17.6%)
	Eastern	26 (6.1%)
	Central	39 (9.2)
Educational level	Literate (able to read and write)	29 (6.8%)
	Below university level (elementary, intermediate, secondary)	82 (19.3%)
	University graduate (Bachelor’s degree) or higher (Master’s, Doctorate)	314 (73.9%)
Occupation	Unemployed	176 (41.4%)
	Employed	230 (54.1%)
	Retired	19 (4.5%)
Monthly income	<5000 SR	155 (36.5%)
	5000–10,000 SR	113 (26.6%)
	>10,000 SR	157 (36.9%)
Residence	House (Villa)	126 (29.6%)
	Apartment	135 (31.8%)
	Family house	164 (38.6%)
Does your work relate to eye health?	Yes	55 (12.9%)
	No	370 (87.1%)

Data are presented as frequencies (*n*) and proportion (%).

**Table 2:** Parents’ knowledge of eye care for their children (total response = 370).

Knowledge of Parents about Eye Care	Category	Frequency and Proportion, *n* (%)
Does your child suffer from any eye conditions?	No eye disease is present	242 (65.4%)
	Lazy eye (amblyopia)	28 (7.6%)
	Refractive error (near or farsightedness)	62 (16.8%)
	Eye allergy	2 (0.5%)
	Strabismus	7 (1.9%)
	Congenital cataract	3 (0.8%)
	Others	26 (7%)
If your child has a vision problem, will they go to school?	No eye disease is present	242 (65.4%)
	Yes, he suffers from a vision problem and still goes to school	122 (33%)
	Yes, he suffers from a vision problem and does not go to school	6 (1.6%)
Why is your child, who suffers from a vision problem, not allowed to go to school?	No eye disease is present	242 (65.4%)
	He cannot learn well without a clear vision	37 (10%)
	The child may be bullied by their peers	19 (5.1%)
	Eye strain due to concentration	1 (0.3%)
	Fearful that the child won’t adapt	16 (4.3%)
	He wears glasses	3 (0.8%)
	Don’t know the cause	52 (14.1%)
What is the source of your information about eye diseases?	Relatives and friends	45 (12.2%)
	Social media	65 (17.5%)
	Internet	92 (24.9%)
	Television	9 (2.4%)
	All mentioned media tools	159 (43%)
The preferred means of disseminating information about children’s eye health	Through doctors	89 (24.1%)
	Awareness campaigns	20 (5.4%)
	Social media	40 (10.8%)
	Internet	14 (3.8%)
	Broadcasting Board	2 (0.5%)
	Television	1 (0.3%)
	All	204 (55.1%)

Data are presented as frequencies (*n*) and proportion (%).

**Table 3:** Knowledge of parents about different eye problems that may affect the child (*n* = 370).

Knowledge of Parents about Different Eye Problems	Category	Frequency and Proportion, *n* (%)
The definition of amblyopia	Decreased vision in one or both eyes	195 (52.7%)
	Degeneration of optic nerve	33 (8.9%)
	Decrease night vision	3 (0.8)
	Misalignment of both eyes	18 (4.9%)
	I don’t know	121 (32.7%)
Causes of amblyopia	Refractive error	52 (14.1%)
	Genetic factors	39 (10.5%)
	Televisions and smart devices	64 (17.4%)
	Strabismus	48 (13%)
	Fever	2 (0.5%)
	Don’t know	165 (44.5%)
Treatment of amblyopia	Patching the strong eye	67 (18.1%)
	Glasses	35 (9.5%)
	Surgery and laser	14 (3.8%)
	Eye muscle exercise	14 (3.8%)
	No need for treatment	2 (5%)
	Patching and glasses	31 (8.4%)
	All options	73 (19.7%)
	I don’t know	134 (36.2%)
In particular, does early treatment of amblyopia lead to better results?	Yes	275 (74.3%)
	No	9 (2.4%)
	I don’t know	86 (23.3%)
Complication of amblyopia	Decreased visual acuity	127 (34.3%)
	Blindness	14 (3.8%)
	Disability	13 (3.6%)
	Impaired quality of life	9 (2.4%)
	School failure	1 (0.3%)
	All the complications mentioned above	76 (20.5%)
	I don’t know	130 (35.1%)
What is a cataract?	A white spot in the eye	53 (14.3%)
	A lens changes where the lens becomes opaque	87 (23.5%)
	A white membrane growing over the eye	50 (13.5%)
	I don’t know	180 (48.7%)
Do cataracts affect children?	Yes	251 (67.8%)
	No	0 (0%)
	Don’t know	119 (32.2%)
Can cataracts lead to permanent blindness in children?	Yes	227 (61.4%)
	No	143 (38.6%)
	I don’t know	0 (0%)
What is glaucoma?	High pressure in the eye	87(23.5%)
	Damage to the nerve of the eye due to high pressure	64 (17.3%)
	An age-related process leading to a decrease in peripheral vision	54 (14.6%)
	I don’t know	165 (44.6%)
Can glaucoma affect children?	Yes	160 (43.2%)
	No	0 (0%)
	I don’t know	210 (56.8%)
Can congenital glaucoma lead to blindness?	Yes	155 (41.9%)
	No	0 (0%)
	I don’t know	215 (58.1%)
How frequently should a child have a routine eye exam?	Every year	185 (50%)
	Every two years	58 (15.7%)
	Every five years	15 (4.1%)
	Only when the child reports a problem	50 (13.5%)
	I don’t know	62 (16.7%)
Visual signs that may prompt you to take your child to an eye care specialist	Tilting the head to one side	9 (2.4%)
	Rubbing the eyes frequently	31 (8.4%)
	Double vision	13 (3.5%)
	Redness of the eye	27 (7.3%)
	Playing with toys from a close distance	36 (9.7%)
	Staring eyes	13 (3.5%)
	Excessive production of tears	3 (0.8%)
	All the above-mentioned	238 (64.4%)
Does any of your children wear glasses? And for what purpose?	None of the children wear glasses	193 (52.2%)
	Yes, lazy eye	37 (10%)
	Yes, refractive errors (nearsightedness, far-sightedness)	100 (27%)
	Yes, strabismus (crossed eyes)	14 (3.8%)
	Yes, not sure of the purpose	26 (7%)
Have you considered the idea of your child wearing contact lenses?	Yes	60 (16.2%)
	No	310 (83.8%)
Would you allow your child to undergo eye surgery if it were necessary?	Yes	300 (81.1%)
	No, fear of the outcome	31 (8.4%)
	No, not sure	25 (6.8%)
	No, cultural and social barriers	10 (2.7%)
	No, the cost of the operation	3 (0.7%)
	No, complications to the eye	1 (0.3%)

Data are presented as frequencies (*n*) and proportion (%).

**Table 4:** Factors associated with parents’ awareness regarding pediatric eye disease.

Factors		Excellent/Good Performance	Low	*p*-Value
Sex	Male	24 (44.4%)	30 (55.6%)	0.392
	Female	121 (38.3%)	195 (61.7%)	
Age	18–40 years	93 (37.2%)	157 (62.8%)	0.521
	>40–60 years	50 (43.5%)	65 (56.5%)	
	>60 years	2 (40%)	3 (60%)	
Residence area	Northern	81 (39.5%)	124 (60.5%)	0.396
	Southern	13 (35.1%)	24 (64.9%)	
	Western	21 (31.8%)	45 (68.2%)	
	Eastern	11 (45.8%)	13 (54.2%)	
	Central	19 (50%)	19 (50%)	
Educational level	Literate (able to read and write)	9 (45%)	11 (55%)	0.622
	Below university level (elementary, intermediate, secondary)	25 (34.7%)	47 (65.3%)	
	University graduate (Bachelor’s degree) or higher (Master’s, Doctorate)	111 (39.9%)	167 (60.1%)	
Occupation	Unemployed	54 (34%)	105 (66%)	0.202
	Employed	84 (43.1%)	111 (56.9%)	
	Retired	7 (43.8%)	9 (56.3%)	
Monthly income	<5000 SR	45 (33.8%)	88 (66.2%)	0.258
	5000–10,000 SR	43 (40.6%)	63 (59.4%)	
	>10,000 SR	57 (43.5%)	74 (56.5%)	
Living place	House (Villa)	41 (39%)	64 (61%)	0.413
	Apartment	43 (35%)	80 (65%)	
	Family house	61 (43%)	81 (57%)	
What is the source of your information about eye diseases?	Relatives and friends	21 (46.7%)	24 (53.3%)	0.393
	Social media	20 (30.8%)	45 (69.2%)	
	Internet	33 (35.9%)	59 (64.1%)	
	Television	4 (44.4%)	5 (55.6%)	
	All mentioned	67 (42.1%)	92 (57.9%)	
The preferred means of disseminating information about children’s eye health	Through doctors	33 (37.1%)	58 (62.9%)	0.138
	Awareness campaigns	10 (50%)	10 (50%)	
	Social media	13 (32.5%)	27 (67.5%)	
	Internet	1 (7.1%)	13 (92.9%)	
	Broadcasting Board	1 (50%)	1 (50%)	
	Television	0 (0%)	1 (100%)	
	All the above-mentioned	87 (42.6%)	117 (57.4%)	
Having a child with eye problems	No eye disease is present	73 (30.3%)	169 (69.7%)	0.0001
	amblyopia	18 (64.3%)	10 (35.7%)	
	Refractive error	36 (58. 1%)	26 (41.9%)	
	Eye allergy	1 (50%)	1 (50%)	
	Strabismus	2 (28.6%)	5 (71.4%)	
	Congenital cataract	1 (33.3%)	2 (66.7%)	
	Others	14 (53.8%)	12 (46.2%)	

Pearson’s chi-square and exact probability tests were applied. The statistical significance was set at *p*-value < 0.05.

**Table 5:** Predictors associated with parents’ knowledge regarding pediatric eye diseases.

Variables	Wald	df	Sig.
Sex	0.124	1	0.725
Age	0.422	2	0.81
Living area	2.062	4	0.724
Educational level	0.853	2	0.653
Occupation	0.368	2	0.832
Monthly income	0.108	2	0.948
Residence	1.45	2	0.484
Having a child with eye problems	21.605	7	0.003
The source of information about eye diseases	6.31	6	0.389

Wald: A statistical test that determines the significance of each coefficient in the model; df: degrees of freedom; Sig.: significance; statistical significance was set at *p*-value < 0.05.

**Table 6:** Relationship between the parents’ knowledge and their practices regarding pediatric eye diseases.

Practices		Overall Knowledge Level		
		Poor	Good/Excellent	*p*-Value
What is the frequency of a child’s routine eye exam?	Every year	108	30	0.001
		48.00%	20.70%	
	Every two years	43	59	
		19.10%	40.70%	
	Every five years	40	28	
		17.80%	19.30%	
	Only when the child reports a problem	19	15	
		8.40%	10.30%	
	I don’t know	15	13	
		6.70%	9.00%	
When the child has an eye problem?	Tilting the head to one side	5	4	0.366
		2.20%	2.80%	
	Rubbing the eyes frequently	17	14	
		7.6%	9.7%	
	Double vision	8	5	
		3.6%	3.4%	
	Redness of the eye	19	17	
		8.4%	11.7%	
	Playing with toys from a close distance	22	5	
		9.8%	3.4%	
	Staring eyes	7	6	
		3.1%	4.1%	
	Excessive tearing	1	2	
		0.4%	1.4%	
	All the above-mentioned	146	92	
		64.90%	63.40%	
Does your child wear glasses?	None of the children wear glasses	136	57	0.0001
		60.40%	39.30%	
	Yes, lazy eye	16	21	
		7.10%	14.50%	
	Yes, refractive errors (nearsightedness, far-sightedness)	46	54	
		20.40%	37.20%	
	Yes, strabismus (crossed eyes)	7	7	
		3.10%	4.80%	
	Yes, not sure of the purpose	20	6	
		8.90%	4.10%	
Do you accept the child wearing contact lenses?	Yes	26	34	0.002
		11.60%	23.40%	
	No	199	111	
		88.40%	76.60%	
Would you allow your child to undergo eye surgery if necessary?	Yes	22	9	0.254
		9.80%	6.20%	
	No, fear of the outcome	18	7	
		8.00%	4.80%	
	No, not sure	174	126	
		77.30%	86.90%	
	No, cultural and social barriers	3	0	
		1.30%	0.00%	
	No, the cost of the operation	7	3	
		3.10%	2.10%	
	No, complications to the eye	1	0	
		0.40%	0.00%	

Pearson’s chi-square and exact probability tests were applied. The statistical significance was set at *p*-value < 0.05.

**Table 7:** Summary of studies in Saudi Arabia concerning assessment of parents’ knowledge and attitude toward pediatric eye disease.

Figure	Study Location	Sample Size	Data Collection	Reported Outcome (s)
Al-Lahim/ 2018 [34]	Tabuk	397	self-administered online	Among participants, 77.6% were female and 41.4% aged between 18 and 25. Half demonstrated sufficient knowledge of common eye issues, primarily informed by the internet (46.7%), relatives (38.5%), and mass media (35.4%). Awareness varied, with 66.3% knowledgeable about cataracts and 36.3% about refractive errors, with education and occupation being significant knowledge determinants. Most respondents (75%) sought an ophthalmologist’s care only for specific complaints; a mere 10% had regular check-ups. When experiencing eye problems, 63% directly consulted an ophthalmologist, while others chose no treatment (9%) or home remedies (7.4%).
Alshail/2018 [35]	Jeddah	678	self-administered questionnaire	In total, 678 participants took part in the survey. Nearly 40% were unsure if cataracts could affect children, and while most could not pinpoint risk factors and symptoms, 75.2% were aware that the condition is treatable, with 46.9% recognizing surgery as the primary treatment. The researchers concluded that limited awareness of pediatric cataracts among Jeddah’s population highlights the need for educational initiatives to increase knowledge.
Parrey/2019 [23]	Arar	1986	personal interview for 10 to 15 min with every parent	While 56.7% of participants had a sufficient understanding of children’s eye disease (CED), knowledge varied significantly with demographic factors. High awareness of refractive errors was noted; 85.6% viewed CED as a severe concern and 73% supported spectacle use for their children. Interest in educational programs on CED was low, with only 26.6% willing to participate. Approximately 68% were satisfied with CED healthcare services, with eye deviation most likely to prompt medical action. Only a third of parents reported routine eye check-ups for their offspring. Advice from family and friends was a leading source of CED information (36.9%). The study concludes a crucial need to improve parental knowledge of CED in Arar through targeted educational efforts, emphasizing early detection and management.
Suratti/2022 [22]	Madinah	555	self-administered questionnaire was randomly distributed	Regarding children’s eye health, only a few parents (3.6%) showed excellent knowledge, while a vast majority (78.2%) had poor understanding. Specific awareness was slightly higher for amblyopia but much lower for childhood cataracts and glaucoma. Most parents favored kids wearing glasses and eye surgery if needed. More than half had taken their kids for an eye check-up. Parents typically gained information from doctors, media campaigns, and social media. Notably, older participants, those with higher incomes, those of Saudi descent, and those with a child with an eye condition demonstrated better knowledge.
Alrasheed/2022 [36]	Saudi Arabia	358	online questionnaire	Around 38.3% of parents were unfamiliar with refractive errors, and 33.8% did not believe uncorrected errors could impair vision. Most parents (74.0%) recognized eyeglasses as an effective management tool for childhood refractive errors. However, approximately 63.7% reported receiving no information about child eye care. Greater awareness was linked to higher education levels, being female, and advancing age. Concerning attitudes toward spectacles, 13.7% of parents thought spectacles could hinder their children’s learning, but 82.7% disagreed that glasses would negatively impact future employment. Notably, 22.1% believed that eyeglass use could weaken the eyes and lead to vision issues in children.
Aldhabaan/2022 [37]	Aseer	899	online questionnaire	Nearly half of the participants had taken their children for eye check-ups. Additionally, 65% were informed about clinics offering eye exams, and 63.3% understood that children with visual impairments could be educated. Overall, more than a third of the parents demonstrated awareness of pediatric eye health. The findings indicate parental awareness and proactive behavior in seeking eye care for their children. However, there is a need for further targeted awareness campaigns to help parents address concerns and barriers to accessing pediatric eye care services.
Alkalash/2023 [9]	Al-Qunfudah	403	self-administered online questionnaire	Parental understanding and perceptions surrounding their children’s eye care were less than ideal, with most eye exams for children prompted by observable eye issues or symptoms. More favorable attitudes towards eye health were observed among parents in the 36–45 age range, those employed, and those with a more significant number of children experiencing eye problems. Additionally, knowledge was more comprehensive among individuals aged 36–45 with at least a bachelor’s degree.
Almogbel/2023 [20]	Makkah	470	self-administered questionnaire was randomly distributed	Most (72.8%) of the surveyed participants (*n* = 470) displayed a limited understanding of children’s eye diseases, while only 2.8% had an excellent grasp of the topic. Eye redness was the most recognized symptom, necessitating a visit to an eye doctor. Additionally, more than two-thirds (68.5%) were open to the possibility of their children undergoing eye surgery, but those against it (11.3%) mainly feared surgical complications.
AlJarallah/2024 [38]	Riyadh	417	online questionnaire	Most participants understood amblyopia as “poor vision in one or both eyes” (19.1%), recognizing the importance of eye coordination. Just over half of parents were aware of lazy eye, primarily informed by the internet and by doctors. Only a small fraction (8.9%) knew that amblyopia is generally untreatable after age 10. Causes associated with amblyopia were mainly identified as genetic and due to refractive errors. Parental awareness levels were notably affected by sociodemographics like gender and education, a family history of eye disease, and having a child with a lazy eye.

## Data Availability

All generated data in this study are included in the article.
